# Therapeutic Potential of Phlorotannin-Rich *Ecklonia cava* Extract on Methylglyoxal-Induced Diabetic Nephropathy in In Vitro Model

**DOI:** 10.3390/md20060355

**Published:** 2022-05-27

**Authors:** Chi-Heung Cho, Chang-Jun Lee, Min-Gyeong Kim, Bomi Ryu, Jun-Geon Je, Yoonsook Kim, Sang-Hoon Lee

**Affiliations:** 1 Division of Functional Food Research, Korea Food Research Institute, Wanju 55365, Korea; chiheungcho@kfri.re.kr (C.-H.C.); 50040@kfri.re.kr (C.-J.L.); 50034@kfri.re.kr (M.-G.K.); kimyus@kfri.re.kr (Y.K.); 2 Department of Food Science and Technology, Chonbuk National University, Jeonju 54896, Korea; 3 Department of Food Biotechnology, University of Science and Technology, Daejeon 34113, Korea; 4 Marine Science Institute, Jeju National University, Jeju 63333, Korea; ryu.bomi@gmail.com (B.R.); wpwnsrjs@naver.com (J.-G.J.)

**Keywords:** *Ecklonia cava*, phlorotannin, diabetic nephropathy, advanced glycation end-products, methylglyoxal, mouse glomerular mesangial cell, RAGE, apoptosis, Nrf2/ARE signaling pathway, MAPK signaling pathway

## Abstract

Advanced glycation end-products (AGEs) play a vital role in the pathogenesis of diabetic complications. Methylglyoxal (MGO), one of the major precursors of AGEs, is a highly reactive dicarbonyl compound that plays an important role in the pathogenesis of diabetic nephropathy. This study was designed to evaluate the therapeutic potential of phlorotannin-rich *Ecklonia cava* extract (ECE) on MGO-induced diabetic nephropathy in in vitro models using mouse glomerular mesangial cells. ECE showed anti-glycation activity via breaking of AGEs-collagen cross-links and inhibition of AGEs formation and AGE-collagen cross-linking formation. The renoprotective effects were determined by assessing intracellular reactive oxygen species (ROS) and MGO accumulation, cell apoptosis, and the Nrf-2/ARE signaling pathway. MGO-induced renal damage, intracellular ROS production level, and MGO-protein adduct accumulation were significantly decreased by pretreating ECE. Moreover, ECE pretreatment exhibited preventive properties against MGO-induced dicarbonyl stress via activation of the Nrf2/ARE signaling pathway and reduction of RAGE protein expression in mouse glomerular mesangial cells. Collectively, these results indicated potential anti-glycation properties and prominent preventive effects of ECE against MGO-induced renal damage. Additionally, ECE may be utilized for the management of AGE-related diabetic nephropathy.

## 1. Introduction

Diabetic nephropathy is one of the serious complications of chronic diabetic conditions. It is known to be one of the leading causes of death in diabetic patients and is a contributing cause of end-stage renal disease [1,2]. Advanced glycation end-products (AGEs) are developed endogenously by non-enzymatic reactions of sugars with amino groups in proteins and lipids. Heat treatment of foodstuffs is a major contributor to exogenous AGE formation. Approximately 10% of AGE molecules are produced in heat-treated foodstuffs and absorbed into the human body, where they accumulate in tissues [3]. They play an important role in the development of AGEs-related diabetic complications [4,5]. In particular, chronic hyperglycemia condition accelerates the rate of AGE formation, which is considered the major cause of diabetic complications such as heart disease, retinopathy, neuropathy, and nephropathy [6,7]. Therefore, the mechanism of inhibiting the generation of AGEs, inhibiting the formation of cross-linking between the AGEs-proteins, and breaking the cross-links between the already produced AGEs-proteins, is considered an effective strategy for preventing and delaying diabetic complications caused by the AGEs. Synthetic drugs, such as aminoguanidine (AG) and Alagebrium (ALT-711), which have the effect of inhibiting the formation of AGEs, inhibiting cross-linking formation, and breaking the generated cross-links were attempted to be developed as therapeutic agents for diabetic complications, but serious side effects were found during clinical trials [8,9,10,11].

AGEs interact with receptors for AGEs (RAGEs) and play a role in the pathogenesis of diabetic complications. Moreover, the interaction between AGEs and RAGEs triggers signaling cascade events and causes damage of various tissues through oxidative stress and apoptotic cell death, namely by reducing basement membrane deformability, degradation, and matrix cell interaction [1,12]. In addition, AGEs accumulate in the glomerular basement membrane and mesangial cells, forming irreversible cross-links with tissue proteins [13]. Methylglyoxal (MGO) is a major precursor of AGE formation, and it is a highly reactive and toxic compound. MGO-induced dicarbonyl stress increases oxidative stress and induces apoptosis by promoting the mitochondrial apoptosis pathway [14,15]. The nuclear factor erythroid 2-related factor 2 (Nrf2) is a critical regulator of genes containing an antioxidant responsive element (ARE) such as catalase (CAT), superoxide dismutase (SOD), heme oxygenase 1 (HO-1), and NAD(P)H: quinone oxidoreductase 1(NQO1) [16,17]. Nrf2 translocates to the nucleus and binds to AREs, activating expression of ARE-dependent genes including the phase II detoxification and antioxidant enzymes [18,19].

*Ecklonia cava*, an edible brown alga, is a richer source of phlorotannins. In particular, several researchers have demonstrated that bioactive compounds contained in *E. cava* are effective in improving and preventing diabetes mellitus (type I and type II) through various mechanisms, such as activating the AMPK and Akt signaling pathways, enhancing glucose and lipid metabolism, and upregulating endogenous antioxidant enzymes [20,21,22]. Although many studies have been conducted on the diverse biological activities of *E. cava*, especially its antidiabetic activity, its preventive effect against AGEs-related diabetic complications has not been investigated.

In the present study, we evaluated the anti-glycation properties of *E. cava* against various mechanisms such as inhibition of AGEs formation, inhibition of AGE-collagen cross-linking formation, and breaking of AGE-collagen cross-links. Additionally, we examined the protective effects of *E. cava* against AGE-induced renal damage in glomerular membrane mesangial cells. Furthermore, the effects of *E. cava* on signaling molecules, such as RAGE, Nrf2/ARE, and MAPK, in the detoxification cascade associated with MGO-induced oxidative stress, were confirmed by Western blotting.

## 2. Results

### 2.1. Phlorotannin Composition in E. cava

**Figure 1:**
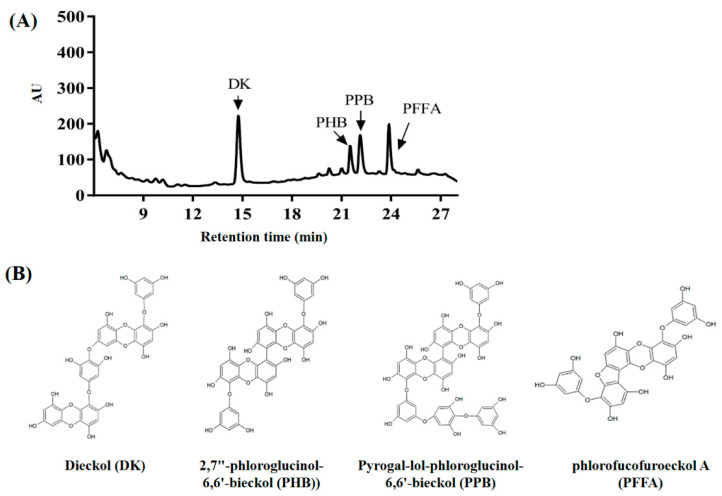
HPLC chromatogram (**A**) of the *Ecklonia cava* extract (ECE). Structure of phlorotannin compounds on the ECE (**B**). DK, dieckol; PHB, 2,7″-phloroglucinol-6,6′-bieckol; PPB, pyrogallol-phloroglucinol-6,6′-bieckol; PFFA, phlorofucofuroeckol A.

According to the chromatogram of *E. cava*, the dominant hydrophilic peaks represented dieckol (DK), 2,7″-phloroglucinol-6,6′-bieckol (PHB), pyrogallol-phloroglucinol-6,6′-bieckol (PPB), and phlorofucofuroeckol A (PFFA) (Figure 1). The chromatogram peaks of each phlorotannin was determined systemically by comparing these results with those of a previous study [23]. The following phlorotannins in the form of organic polymers were contained in *E. cava*: phloroglucinol (1,3,5-trihydroxybenzene), DK (742.07 g/mol, 14.8 min), PHB (974.11 g/mol, 21.5 min), PPB (974.14 g/mol, 22.2 min), and PFFA (602.06 g/mol, 24.0 min.

### 2.2. Anti-Glycation Ability of E. cava

**Figure 2:**
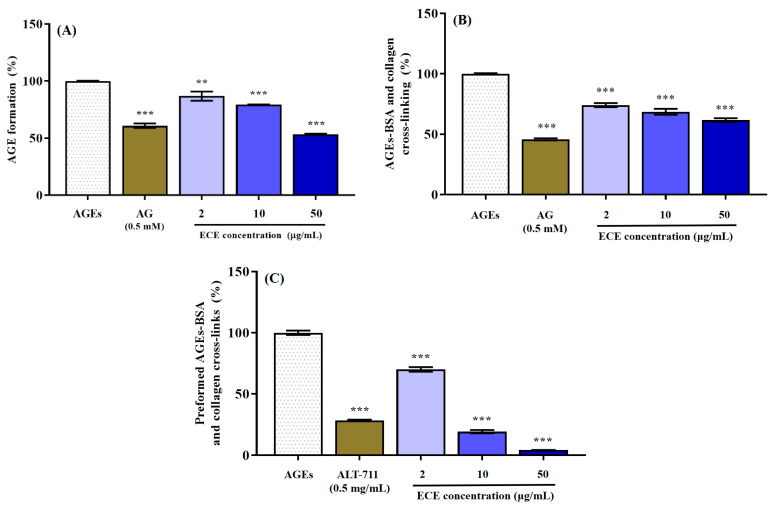
Effects of the *Ecklonia cava* (ECE) extract on AGE-induced glycation reaction in vitro. Inhibition of AGE formation (**A**), inhibition of AGEs-BSA and collagen cross-link formation (**B**), and breaking of AGEs-BSA and collagen cross-links (**C**). Data are expressed as mean ± standard deviation (*n* = 6) and statistical analysis of the results was performed using one-way ANOVA followed by Tukey’s test (** *p* < 0.01 and *** *p* < 0.001 vs. AGEs).

The ability of ECE to inhibit the AGEs formation is presented in Figure 2A. The inhibitory effect of ECE on AGEs formation was evaluated at concentrations of 2, 10, and 50 μg/mL, and 0.5 mM aminoguanidine was used as a positive control. ECE pretreatment significantly suppressed the AGEs formation at all tested concentrations (*** *p* < 0.001), and 50 μg/mL of ECE pretreatment reduced AGEs formation by 53.35 ± 1.07% compared to the control. In particular, 50 μg/mL of ECE pretreatment exhibited a higher inhibitory ability on AGEs formation than aminoguanidine (60.87 ± 3.27%), a synthetic agent that inhibits the generation of AGEs.

The effect of ECE in inhibiting cross-linking generation between AGEs-collagen is shown in Figure 2B. ECE exhibited significant activity in inhibiting the generation of cross-linking between AGEs-collagen at all tested concentrations, and when 50 μg/mL ECE was treated (*** *p* < 0.001), it showed the highest cross-links formation inhibitory effect by 61.72 ± 2.66% compared to the control (nontreated group). In the case of AG, which is known as a synthetic inhibitor that inhibits the cross-linking between AGEs-collagen, the cross-linking generation inhibitory efficacy was 45.83 ± 1.43% at a concentration of 0.5 mM.

We evaluated the cross-link breaking ability of ECE on cross-links formed between AGEs-collagen using ELISA (Figure 2C). ECE pretreatment exhibited the breaking ability of the cross-links formed between AGEs-collagen in a concentration-dependent manner at all treated concentrations (*** *p* < 0.001). In particular, 50 μg/mL ECE pretreatment dramatically reduced the cross-links formed between AGEs-collagen to 4.25 ± 0.41% and showed superior ability to ALT-711 (28.49 ± 1.05%), which is a known representative cross-links breaker. Taken together, our results indicated that ECE effectively inhibited AGEs, one of the causes of accelerated diabetic complications, through a variety of mechanisms. In addition, ECE is considered to have a higher potential than synthetic drugs developed for prevention of AGEs-related diabetic complication, such as AG and ALT-711.

### 2.3. Protective Effects of E. cava against MGO-Induced Renal Damage

**Figure 3:**
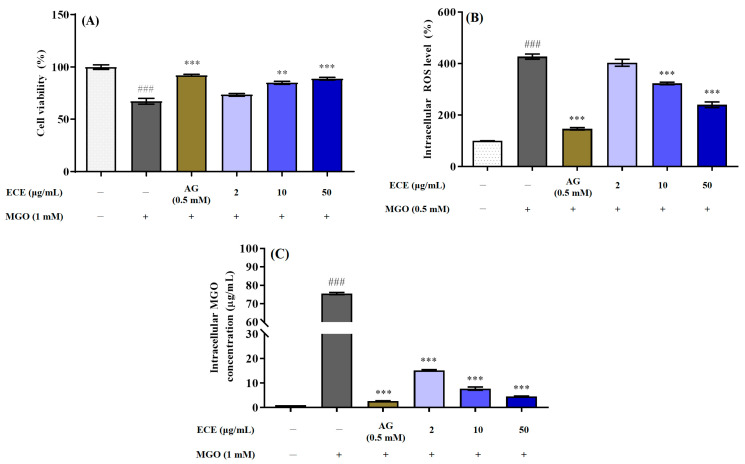
Effects of the *Ecklonia cava* extract (ECE) against MGO-induced renal damage in mesangial cells. Protective effect of ECE against MGO-induced oxidative stress in mesangial cells was evaluated by the MTT reduction assay (**A**). Intracellular reactive oxygen species (ROS) scavenging activity of ECE against MGO-induced oxidative stress in mesangial cells was determined by the DCFH-DA assay (**B**). Inhibitory effect of ECE on AGE-induced intracellular MGO-protein adduct accumulation was analyzed using the MGO Competitive ELISA kit (**C**). Data are expressed as mean ± standard deviation (*n* = 6) and statistical analysis of the results was performed using one-way ANOVA followed by Tukey’s test (^###^ *p* < 0.001 vs. control, ** *p* < 0.01, and *** *p* < 0.001 vs. MGO only).

Prior to verifying the renoprotective effect of ECE on MGO-induced renal damage, the cytotoxicity of ECE was evaluated using the MTT assay. A reduction in cell viability to less than 80% compared with the control group was considered to be cytotoxic. All treated concentrations of ECE (2, 10, and 50 μg/mL) were noncytotoxic to mesangial cells (data not shown). MGO treatment significantly (^###^ *p* < 0.001) reduced the mesangial cells viability to 67.21 ± 4.90% compared to the control group (nontreated cells) (Figure 3A). On the other hand, pretreatment with ECE significantly increased cell viability in a concentration-dependent manner compared to MGO only treated cells (** *p* < 0.01, *** *p* < 0.001 vs. only MGO treated cells). In particular, 50 μg/mL ECE pretreatment increased the cell viability to 88.77 ± 2.21%, exhibiting the highest cytoprotective effect against MGO-induced renal damage.

The DCFH-DA assay was performed to examine the intracellular antioxidant capacity of ECE against MGO-induced oxidative stress in mesangial cells (Figure 3B). Treatment of 1 mM MGO significantly increased intracellular ROS levels by 427.42 ± 17.29% compared to nontreated cells (^###^ *p* < 0.001). On the other hand, pretreatment with ECE showed the effect of significantly suppressing intracellular ROS generation in concentration-dependent manner in mesangial cells. In particular, the pretreatment with 50 μg/mL ECE exhibited the strongest ROS production inhibitory effect, and it decreased the intracellular ROS generation level to 240.21 ± 19.35% compared with only MGO-treated cells (*** *p* < 0.001).

To quantitatively verify the inhibitory effect of ECE on intracellular MGO accumulation, intracellular MGO quantitative analysis was performed using OxiSelectTM Methylglyoxal (MG) Competitive ELISA kit. Treatment with 1 mM MGO significantly increased the MGO cross-linked protein concentration in mesangial cells to approximately 75.55 ± 1.02 μg/mL when compared to the nontreated cells (1.02 ± 0.01 μg/mL) (^###^ *p* < 0.001). However, pretreatment with ECE dramatically reduced the intracellular MGO accumulation at all tested concentration. Notably, it was confirmed that 50 μg/mL ECE (4.59 ± 0.28 μg/mL) pretreatment decreased the intracellular MGO accumulation to a concentration similar to that of the nontreated cells, and this effect of ECE was the same level as that of 0.5 mM AG (2.70 ± 0.10 μg/mL) (*** *p* < 0.001).

### 2.4. Preventive Effects of E. cava against MGO-Induced Apoptotic Cell Death

**Figure 4:**
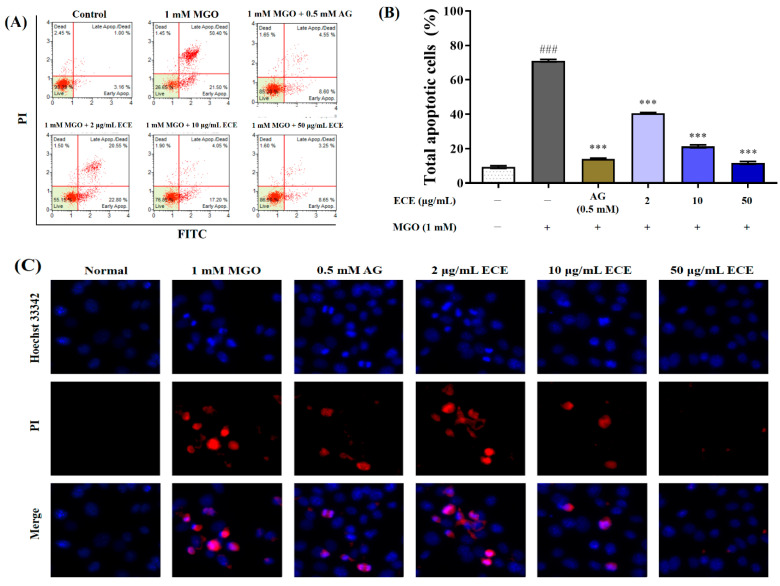
Protective effect of the *Ecklonia cava* extract (ECE) against MGO-induced apoptotic cell death. Annexin V-based apoptotic cell death was measured by MUSE cell analyzer using a Muse™ Annexin V & Dead Cell kit (**A**). The graph represents proportion of early and late apoptotic cells (**B**). Representative images of Hoechst 33342/PI double staining (**C**). Data are expressed as mean ± standard deviation (*n* = 3). Significant differences among the groups were determined by one-way ANOVA followed by Tukey’s test (^###^ *p* < 0.001 vs. control and *** *p* < 0.001 vs. MGO only).

Apoptosis analysis was performed using the Muse™ Annexin V & Dead Cell Kit (Figure 4A). MGO treatment significantly increased the ratio of apoptotic cell death of mesangial cells from 9.39 ± 1.35% to 71.15 ± 1.26% (^###^ *p* < 0.001). However, ECE pretreatment reduced MGO-induced apoptotic cell death in a concentration-dependent manner (*** *p* < 0.001). In particular, when cells were pretreated with 50 μg/mL ECE, apoptotic cell death was significantly suppressed by approximately 11.78 ± 1.43% compared with only MGO treated cells (Figure 4B). AG, used as the positive control, also effectively inhibited the apoptosis of mesangial cells to 14.07 ± 0.81% (*** *p* < 0.001). To further confirm the effects of ECE on MGO-induced apoptotic cell death, mesangial cells were stained with Hoechst 33258/propidium iodide (PI). Hoechst 33258/PI double staining revealed that MGO-treated cells exhibited typical features of apoptotic bodies. ECE pretreatment dramatically reduced the number of apoptotic and necrotic cells (Figure 4C). Hoechst 33258/PI double staining strongly supports these results. These results indicate that ECE has a renoprotective ability to prevent MGO-induced renal damage via intracellular antioxidative activity and inhibition of intracellular MGO accumulation.

### 2.5. Effects of E. cava on RAGE Protein Expression

**Figure 5:**
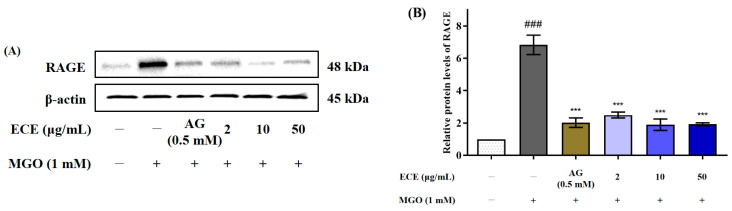
Effect of the *Ecklonia cava* extract (ECE) on protein expression of the receptor for advanced glycation end-products (RAGE) in MGO-induced mesangial cells. Cells were pretreated with ECE for 1 h, followed by incubation with 1 mM MGO for 24 h. The protein expression levels of RAGE were measured by Western blotting (**A**). RAGE band intensity; β-actin was used as an internal control (**B**). Data are expressed as mean ± standard deviation (*n* = 3). Statistical analysis of the results was performed using one-way ANOVA followed by Tukey’s test (^###^ *p* < 0.001 vs. control and *** *p* < 0.001 vs. MGO only).

MGO, known as a precursor of AGEs, is directly involved in ROS generation as well as intracellular signaling through RAGE. In addition, activation of RAGE protein expression induces the activation of inflammatory cytokines, acceleration of angiogenesis, and apoptosis in diverse cells [24]. Therefore, control of RAGE protein expression is considered as one of the important strategies to prevent AGEs-related diabetic complications. The effect of ECE on RAGE protein expression was evaluated by Western blotting (Figure 5A). Nearly an eight-fold increase in the RAGE protein expression was observed in MGO treated mesangial cells compared to nontreated cells (^###^ *p* < 0.001). In contrast, pretreatment with ECE dramatically suppressed MGO-induced RAGE expression at all treated concentrations (*** *p* < 0.001) (Figure 5B). In addition, 10 μg/mL ECE pretreatment the strongest suppressed RAGE protein level close to nontreated cells, and inhibited RAGE protein expression more effectively than AG, known as an AGEs inhibitor.

### 2.6. Effects of E. cava on Nrf2/ARE Signaling Pathway

**Figure 6:**
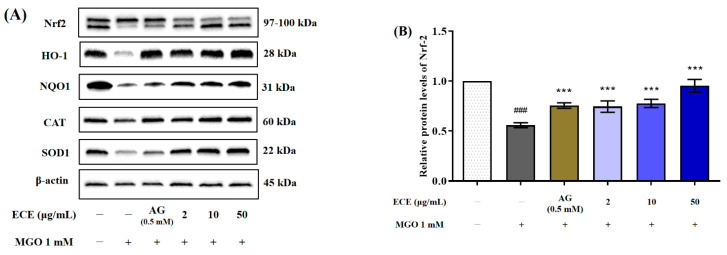

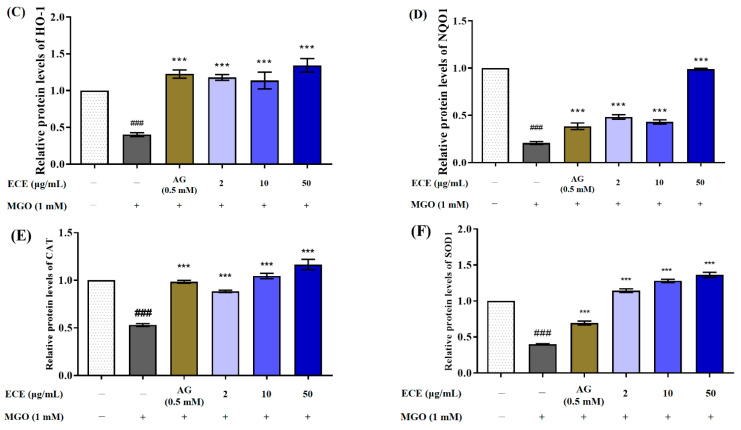
The effect of the *Ecklonia cava* extract (ECE) on antioxidant-related protein expression in MGO-induced mesangial cells. Cell were pretreated with ECE for 1 h, followed by incubation with 1 mM MGO for 24 h. The protein expression levels of Nrf2 and the downstream molecules HO−1, NQO1, CAT, and SOD1 were measured by Western blotting (**A**). Nrf2, HO-1, NQO1, CAT, and SOD1 band intensity; β-actin was used as an internal control (**B**–**F**). Data are expressed as mean ± standard deviation (*n* = 3). Statistical analysis of the results was performed using one-way ANOVA followed by Tukey’s test (^###^ *p* < 0.001 vs. control and *** *p* < 0.001 vs. MGO only).

To confirm the renal protective effect of ECE from MGO-induced oxidative stress, we investigated the effect of ECE on expression of Nrf2 protein and the expression of antioxidant response elements (ARE) such as HO-1, CAT, NQO1, and SOD1, known as downstream regulated genes of Nrf2 (Figure 6A). Treatment with 1 mM MGO extremely reduced the Nrf2 expression level to 0.11-fold compared to nontreated cells (^###^ *p* < 0.001) (Figure 6B). However, pretreatment with ECE significantly inhibited the decrease in Nrf2 protein expression by MGO treatment at all concentrations (2, 10, and 50 μg/mL) (*** *p* < 0.001). In addition, ECE pretreatment suppressed the reduction of Nrf2 protein expression by MGO more effectively than AG. Nrf2 downstream regulated genes expression levels such as HO-1, NQO1, CAT, and SOD1 exhibited reduction by 0.40 (^###^ *p* < 0.001), 0.21 (^###^ *p* < 0.001), 0.49 (^###^ *p* < 0.001), and 0.39-fold (^###^ *p* < 0.001), respectively, compared to nontreated cells, which was also significantly improved by ECE pretreatment (*** *p* < 0.001) (Figure 6C–F). These results indicated that ECE protects mesangial cells against MGO-induced dicarbonyl stress by activating the expression of Nrf2 as well as the expression of downstream pathway phase Ⅱ detoxification and antioxidant enzymes.

### 2.7. Effects of E. cava on MAPK Signaling Pathway

**Figure 7:**
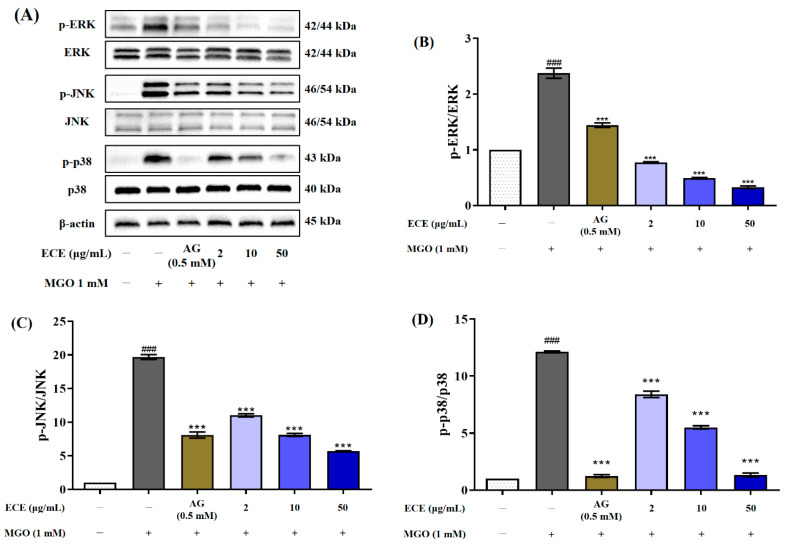
Effect of the *Ecklonia cava* extract (ECE) on the mitogen-activated protein kinase (MAPK) signaling pathway in MGO-induced mesangial cells. Cells were pretreated with ECE for 1 h, followed by incubation with 1 mM MGO for 24 h. The protein expression levels of MAPKs (extracellular signal-regulated kinase; ERK, c-Jun N terminal kinase; JNK, p38, and phosphorylated forms) were determined by Western blotting (**A**). p-ERK/ERK, p-JNK/JNK, and p-p38/p38 ratios (**B**–**D**). Data are expressed as mean ± standard deviation (*n* = 3). Statistical analysis of the results was performed using one-way ANOVA followed by Tukey’s test (^###^ *p* < 0.001 vs. control and *** *p* < 0.001 vs. MGO only).

To verify the protective effect of ECE on apoptotic cell death by MGO-induced oxidative stress, the intracellular MAPKs (ERK, JNK, and p38) signaling pathway was investigated using Western blot (Figure 7A). Treatment with 1 mM MGO significantly induced the phosphorylation of MAP signaling proteins compared to the nontreated cells (Figure 7B–D). However, pretreatment with ECE in mesangial cells dramatically suppressed the phosphorylation of ERK, JNK, and p-38. In particular, it was confirmed that treatment with 50 μg/mL ECE inhibited MAPK phosphorylation more effectively than 0.5 mM AG used as a positive control. In conclusion, ECE plays a role in mediating inhibition of MGO-induced MAPK phosphorylation activation. This result is considered to be due to the action of the phlorotannin compounds contained in ECE lowering the expression of proteins related to apoptotic cell death by MGO-induced oxidative stress. Therefore, we suggest that ECE is a natural resource with the potential to prevent or delay the pathogenesis of AGEs-associated diabetic nephropathy.

## 3. Discussion

*Ecklonia cava*, an edible brown alga, is abundantly distributed along the southern coast of Korea. Previous reports have shown that *E. cava* contains an abundance of a variety of phlorotannin derivatives including eckol, 6,6′-bieckol, dieckol, phlorofucofuroeckol A, 2,7″-phloroglucinol-6,6′-bieckol, and pyrogallol-phloroglucinol-6,6′-bieckol, which exhibit diverse biological activities [25,26]. In addition, phlorotannins are responsible for a variety of biological activities of *E. cava*, including anti-inflammatory, immunomodulatory, neuroprotective, anti-cancer, and anti-diabetic effects [22,27,28]. *E. cava* contains bioactive phlorotannins such as dieckol, pyrogallol-phloroglucinol-6.6-bieckol, 2,7”-phloroglucinol-6,6′-bieckol, and phlorofucofuroeckol-A (Figure 1). Although several reports have suggested that *E. cava* extract (ECE) or phlorotannins isolated from *E. cava* exhibit anti-diabetic effects, there are no reports on the effects of ECE on anti-glycation activity, renoprotective effect on MGO-induced oxidative stress, and the molecular signaling mechanism. Therefore, in this study, the potential anti-glycation and renoprotective effects of *E. cava* on mouse glomerular mesangial cells were investigated.

Advanced glycation end-products (AGEs) are irreversible products generated by a glycation reaction; they are complex heterogeneous molecules that cause protein cross-linking, show browning, and produce fluorescence [7]. This glycation reaction is further increased in diabetic patients with chronic hyperglycemia, accelerating the accumulation of AGEs in various body tissues. Because the accumulation of AGEs affects extracellular proteins and binds to AGEs receptors to activate cytokine production and transcription factors, it has been suggested as a major key in the pathogenesis of AGEs-related diabetic complications. [29]. Therefore, mechanisms to inhibit the formation of AGEs and inhibiting or breaking the cross-linking between AGEs and collagen for suppress the accumulation of AGEs in the body may be effective strategies to prevent and delay AGEs-related diabetic complications. In the present study, it was confirmed that ECE effectively inhibited AGE formation and formation of AGE-collagen cross-linking (Figure 2A,B). In particular, ECE showed an excellent effect in breaking the cross-links between AGEs and collagen (Figure 2C). Therefore, our results suggest that ECE has the potential to be utilized as a powerful natural anti-glycation agent.

MGO, a highly reactive dicarbonyl compound, is a major precursor of AGEs. MGO is generated primarily as a byproduct of several metabolic pathways such as glycolysis, lipid peroxidation and amino acid metabolism. Non-enzymatic degradation of the glycolytic intermediates dihydroxyacetone phosphate (DHAP) and glyceraldehyde 3-phosphate (GA3P) is a major cause of MGO formation. Additionally, threonine catabolism, nonenzymatic decarboxylation and acetoacetate oxidation, and glucose degradation are also known to cause MGO formation. Therefore, MGO is ubiquitous in all biological organisms because it is formed from the spontaneous degradation of endogenous metabolites. The glyoxalase system, which consists of two enzymes, glyoxalase-1 (Glo-1) and Glo-2, is known to play an important role in directly inhibiting the AGEs formation by detoxifying reactive carbonyl compounds such as MGO into nontoxic D-lactate [30,31,32]. However, a sustained increase in the generation of MGO, MGO adducts, and MGO-derived AGEs have been implicated in the development of diabetic complications, such as retinopathy, cardiovascular disease, and nephropathy [15,33]. In particular, oral administration of MGO accumulates collagen in the kidney and causes renal dysfunction due to thickening of the glomerular basement membrane [34]; therefore, detoxification of MGO is considered an important strategy for preventing or suppressing AGE-related nephropathy. Furthermore, several researchers have suggested that natural resource extracts or natural-source-derived polyphenolic compounds effectively act in the prevention of AGE-related diabetic complications through trapping of methylglyoxal, chelating metal ions, scavenging reactive oxygen species, covering the glycation sites of proteins, and regulating RAGE protein expression [35,36]. Here, MGO treatment significantly increased cytotoxicity, intracellular ROS generation, and intracellular MGO- and MGO-derived AGE accumulation. Conversely, ECE pretreatment exhibited a renoprotective effect against MGO-induced renal damage (Figure 3A) and significantly reduced intracellular ROS generation (Figure 3B). Additionally, ECE dramatically suppressed the accumulation of MGO and MGO-derived AGEs in mesangial cells (Figure 3C). Moreover, ECE pretreatment reduced renal apoptotic cell death caused by MGO-induced oxidative stress (Figure 4). Thus, ECE is considered effective in protecting renal damage from MGO-induced oxidative stress by inhibiting intracellular ROS generation and AGE accumulation.

Our results demonstrated that MGO-induced oxidative stress and apoptosis could be significantly reduced by ECE pretreatment, but the mechanism of action is not clear. MGO is a reactive α-oxoaldehyde metabolite that is a known AGE precursor. AGEs initiate a cascade of receptor-dependent pathways, primarily through interaction with the AGEs-specific cell surface receptor RAGE, which is a major cause of endothelial dysfunction by increasing oxidative stress and stimulating pro-inflammatory processes. [37]. AGE molecules bind only to the RAGE V domain. Rather than accelerating or retarding cellular activation, AGE-RAGE interaction initiates sustained cellular activity mediated by receptor-dependent signals that induce inflammation and ROS generation [38]. In addition, MGO-induced protein glycation activates RAGE via ROS generation in mesangial cells [39]. MGO further accelerates ROS production by activating the AGE-RAGE interaction [40]. Therefore, the effect of ECE in preventing apoptosis due to activation of a specific signaling pathway by MGO was confirmed by measuring RAGE protein expression (Figure 5). RAGE protein expression was increased by MGO treatment, whereas pretreatment with ECE suppressed the MGO-induced increase in RAGE protein expression.

AGEs cause deleterious effects on cells by increasing their production of free radicals and related reactive intermediates. In addition, accumulation of AGE-bound proteins causes oxidative stress, triggering inflammatory signaling that contributes to the pathogenesis of metabolic diseases [41]. This suggests that MGO promotes oxidative cellular stress while simultaneously increasing intracellular ROS production. Together, these activities cause ROS-mediated cell damage and apoptosis. In the absence of an inducer, Nrf2 is generally found in the cytoplasm in complex with Kelch-like ECH-associated protein 1 (Keap1). When cells are exposed to oxidative stress and the concentrations of reactive species increase, Nrf2 is released from the Nrf2-keap1 protein complex and translocates to the nucleus, where it binds to AREs inducing expression of antioxidant and detoxification enzymes [42,43,44]. In many previous studies, polyphenol compounds such as phenolic acids, flavonoids, and phlorotannins contained in natural resources play important roles in activating Nrf2. Such bioactive compounds have been suggested to offer an effective route to preventing AGE-related diabetic complications by activating Nrf2 to induce expression of antioxidant enzymes [45,46,47]. Representative antioxidant enzymes CAT, SOD1, HO-1, and NOQ1 are regulated by Nrf2, which can reduce AGE-induced oxidative stress [48]. We hypothesized that ECE would suppress MGO-induced oxidative stress and renal cell damage by activating Nrf2 and increasing ARE signaling. Western blotting was performed to determine whether ECE inhibited the reduction in Nrf2 expression caused by MGO treatment. We found that MGO significantly reduced the expression of Nrf2 protein as well as the expression of antioxidant-related downstream genes in mesangial cells (Figure 6). In contrast, ECE pretreatment significantly increased Nrf2 protein expression, as well as the expression of antioxidant-related enzymes such as HO-1, NQO1, CAT, and SOD1. These results suggest that ECE can mitigate MGO-induced oxidative stress by activating the Nrf2 signaling pathway and reducing ROS generation in mesangial cells.

MGO-induced apoptotic cell death in mouse glomerular mesangial cells is closely related to p38 MAPK activity [49]. Several researchers have reported that interaction between AGE and RAGE triggers molecular signaling pathways including the MAPK signaling pathway [12,50,51]. Many previous studies have shown that MAPK activation is an important signaling mechanism involved in MGO-induced apoptotic cell death [52,53]. With this knowledge, we attempted to clarify the intracellular mechanisms responsible for MGO-induced apoptosis in mesangial cells. MAPK plays an important role in cell differentiation. ERK is associated with the proliferation and progression of certain cellular systems, but along with ERK, JNK and p38 are also associated with apoptosis [54]. MAPKs can be activated independently and are involved in cell death. In recent years, it has been suggested that MGO-induced cytotoxicity is associated with the activation of MAPK family members, including ERK, JNK, and p38 [49,55]. MGO is well known to induce glomerular damage through MAPK activation under diabetic conditions. In addition, MGO treatment induces sufficient cellular oxidative stress to induce apoptosis via p38a MAPK activation in rat mesangial cells. This molecular mechanism may be involved in the development of glomerulosclerosis in diabetic patients [49]. Several previous studies have demonstrated that treatment with botanical resources and their antioxidants, including phenolic and flavonoid compounds, suppresses MAPK activation in MGO-induced cells [53,56,57]. Therefore, suppressing the activation of MAPK signaling, including JNK, p38, and ERK, is thought to be an effective therapeutic strategy for diabetic nephropathy. In this study, phosphorylation of MAPK following MGO treatment was confirmed by Western blotting. However, ECE pretreatment inhibited the activation of ERK, JNK, and p38 in mesangial cells (Figure 7). Inhibition of apoptosis by ECE was accompanied by inhibition of MAPK activation, showing that ECE can regulate the MAPK signaling pathway in MGO-treated mesangial cells. Therefore, our results suggest that ECE has an inhibitory effect on MGO-induced apoptosis by suppressing MAPK phosphorylation while simultaneously regulating the Nrf2 pathway and reducing ROS generation in mesangial cell.

## 4. Materials and Methods

### 4.1. Chemicals and Reagents

Aminoguanidine (AG), Alagebrium (ALT-711), glucose, fructose, bovine serum albumin (BSA), sodium azide, 3,3,5,5-tetramethylbenzidine (TMB) liquid substrate system for enzyme-linked immunosorbent assay (ELISA), methylglyoxal (MGO), sodium phosphate monobasic, and sodium phosphate dibasic were purchased from Sigma (St. Louis, MO, USA). Dulbecco’s modified Eagle’s medium/F-12 Nutrient Mixture (DMEM/F-12; Ham, 3:1 mixture) was obtained from Welgene Inc. (Daegu, Korea). Fetal bovine serum (FBS), hydroxyethyl-piperazineethane-sulfonic acid (HEPES), penicillin/streptomycin solution, and collagen I-coated plates were purchased from Gibco (Rockville, MD, USA). PRO-PREP™ Protein Extraction Solution, the OxiSelect™ Methylglyoxal (MG) Competitive ELISA Kit, and the receptor for advanced glycation end-products (RAGE) antibody were purchased from iNtRON Biotechnology (Seongnam, Korea), Cell Biolabs Inc. (San Diego, CA, USA), and Merck Millipore (Billerica, MA, USA), respectively. Antibodies against catalase (CAT), heme oxygenase 1 (HO-1), NAD(P)H:quinone oxidoreductase 1 (NQO1), extracellular signal-regulated kinases (ERK), phospho-ERK (p-ERK), c-Jun N-terminal kinase (JNK), phosphor-JNK (p-JNK), p38, phosphorp38 (p-p38), and superoxide dismutase 1 (SOD1) were purchased from Cell Signaling Technology (Danvers, MA, USA). β-actin and nuclear factor erythroid 2-related factor (Nrf2) antibodies were obtained from Santa Cruz Biotechnology (Santa Cruz, CA, USA). Dieckol (DK), 2,7′-phloroglucinol-6,6′-bieckol (PHB), pyrogallol-phloroglucinol-6,6′-bieckol (PPB), and phlorofucofuroeckol A (PFFA) were kindly donated by Prof. You-Jin Jeon (Jeju National University).

### 4.2. Extraction

Briefly, *Ecklonia cava* (*E. cava*) was collected in July 2020 from the Jeju coastline (South Korea), desalted, dried, and stored at −20 °C. Dried *E. cava* powder was extracted with 50% (*v*/*v*) ethanol at room temperature for 12 h. The extract was then concentrated and freeze-dried. *E. cava* extracts (ECEs; kindly provided by Prof. YJ Jeon) were sealed and stored at −20 °C.

### 4.3. Identification of Phlorotannins Using HPLC

Chromatographic analyses were conducted using an Acquity Arc system (Waters) equipped with a 2998 PDA detector (Beverly, MA, USA) and an Agilent poroshell 120 EC-C18 column (4.6 mm × 100 mm, 4 µm). Gradient elution was performed with a binary mobile phase (solvent A: water; solvent B: acetonitrile, both containing 0.1% (*v*/*v*) formic acid). The initial mobile phase condition was 37% B, which increased linearly to 50% over 10 min. The column was then held at 65% for 20 min, then decreased to 37% B over 5 min. and held at 37% B for 35 min to equilibrate the column. Flow rate was maintained at 0.3 mL/min, at a column temperature of 40 °C. Sample injection volume was 10 µL for 1 mg/mL ECE prepared as methanolic solutions. The detector was set at 220 nm. Chromatogram peaks were assigned by comparison with four phlorotannins (DK, PHB, PPB, and PFFA) isolated from ECE.

### 4.4. Anti-Glycation Abililty

To measure the AGEs formation inhibitory ability of ECE, 10 mg/mL BSA, 200 mM glucose and fructose mixture and ECE (2, 10, and 50 μg/mL) or AG (0.5 mM) were mixed at 7:1:2. After mixing in the ratio, the reaction was carried out at 37 °C incubator for 7 days. For the control group, 50 mM phosphate buffer (pH 7.4) was added in the same ratio instead of ECE. After 7 days, the AGEs formation level was measured at excitation and emission wavelengths of 350 and 450 nm, respectively, using a microplate reader (Molecular Devices, Sunnyvale, CA, USA) and expressed as a percentage (%) decrease in the fluorescence intensity of the control (without test samples).

The ability of ECE in inhibiting cross-linking formation between AGEs and collagen was evaluated by ELISA. 5 μg/mL horseradish peroxidase (HRP)-labeled AGEs solution and ECE (2, 10, and 50 μg/mL) or AG (0.5 mM) were mixed in a 1:1 ratio and then dispensed into a collagen-coated 96-well plate. For the control (without ECE or AG), 50 mM phosphate buffer (pH 7.4) was added in the same ratio instead of ECE. Afterwards, in order to form cross-linking between AGEs and collagen, it was reacted at 37 °C in an incubator for 18 h. After removing the supernatant, the 96-well plate was washed 3 times using 0.05% PBST (PBS with 0.05% Tween 20). To confirm the cross-linking formed between AGEs and collagen, 100 μL TMB substrate solution was added and the absorbance at 450 nm was measured using a microplate reader (Molecular Devices). Data are expressed as percent decrease in optical density.

ELISA was performed to evaluate the efficacy of ECE to breaking the cross-links formed between AGE-collagen. To form cross-links between AGEs and collagen, 100 μL of 5 μg/mL horseradish peroxidase (HRP)-labeled AGEs solution was dispensed into a collagen-coated 96-well plates and incubated at 37 °C for 4 h. After removing the supernatant, the 96-well plate was washed 3 times using 0.05% PBST (PBS with 0.05% Tween 20). Then, 100 μL of ECE (2, 10, and 50 μg/mL) or ALT-711 (500 μg/mL) was added in the complexes of AGEs-collagen and reacted at 37 °C incubator for 18 h. The unattached AGEs-BSA was washed with 0.05% PBST, and 100 μL of the TMB substrate solution was added. Absorbance was measured at 450 nm using a microplate reader (Molecular Devices). Data are expressed as a percentage (%) decrease in optical density.

### 4.5. Cell Culture

Mouse glomerular mesangial cells (mesangial cells) was purchased from ATCC (CRL-1927^TM^, Rockville, MD, USA) and cultured in a 3:1 mixture of DMEM/F-12 medium containing 5% FBS, 14 mM HEPES, 100 U/mL penicillin, and 100 μg/mL streptomycin at 37 °C in 5% CO_2_ and 95% air.

### 4.6. Assessment of Cell Viability

Mesangial cells were seeded in 96-well plates (3.0 × 10^4^ cells/well) using DMEM/F-12 with 5% FBS. Then, pre-incubation was carried out at 37 °C for 6 h. After removal of the supernatant, the cells were pretreated with different concentrations (2, 10, and 50 μg/mL) of ECE for 1 h, followed by treatment with 1 mM MGO for 24 h. After removing the supernatant, 5 mg/mL MTT reagent was added and incubated at 37 °C for 4 h. Absorbance was measured using a microplate reader (Infinite M200; Tecan Austria GmbH, Grödig, Austria) at 570 nm (detection wavelength) and 630 nm (reference wavelength). Cell viability was expressed as a percentage (%) the absorbance of the control cells (without ECE or MGO).

### 4.7. Determination of Intracellular ROS Production Level

Mesangial cells were incubated in 96-well plates (3.0 × 10^4^ cells/well) using DMEM/F-12 with 5% FBS. Then, pre-incubation was carried out at 37 °C for 6 h. After removal of the supernatant, the cells were pretreated with different concentrations (2, 10, and 50 μg/mL) of ECE for 1 h, followed by treatment with 1 mM MGO for 24 h. The cells were washed twice with HBSS and incubated with 10 μM DCFH-DA in HBSS for 30 min. Fluorescence intensity was measured using a microplate reader (Infinite M200; Tecan Austria GmbH) at excitation and emission wavelengths of 485 and 530 nm, respectively. Results are expressed as percent decrease in fluorescence intensity relative to the control (without ECE or MGO).

### 4.8. Determination of Intracellular MGO-Derived AGEs Concentration

Accumulation of intracellular MGO-derived AGEs were evaluated using the ELISA. Mesangial cells were seeded into 6-well plates (1.0 × 10^6^ cells per well) using DMEM/F-12 with 5% FBS and pre-incubated for 6 h. After removing the supernatant, the cells were pretreated with various concentrations of ECE (2, 10, and 50 μg/mL) for 1 h, followed by treatment with 1 mM MGO for 24 h. The cells were collected and lysed to measure the intracellular MGO-derived AGE concentrations using the OxiSelect™ Methylglyoxal Competitive ELISA kit. The absorbance was determined using a microplate reader (Infinite M200; Tecan Austria GmbH) at 450 nm.

### 4.9. Hoechst 33342/PI Double Stanining

Mesangial cells were seeded into 24-well plates (5.0 × 10^5^ cells/well) using DMEM/F-12 with 5% FBS and pre-incubated for 6 h. After removing the supernatant, cells were treated with various concentrations (2, 10, and 50 μg/mL) of ECE for 1 h, followed by treatment with 1 mM MGO for 24 h. Cells were then washed three times with ice-cold PBS, immediately fixed in 4% formalin for 10 min, and permeabilized with 0.2% Triton X-100 for 10 min. Cells were then washed three times with PBS and stained with 2 μg/mL Hoechst 33258 or 10 μg/mL PI staining solution at 37 °C for 30 min. The images were acquired using a fluorescence microscope (Zeiss Aio Observer A1, ZEISS, Jena, Germany).

### 4.10. Apoptosis Analysis

Induction of apoptosis was assessed using a Muse™ Annexin V & Dead Cell Kit (Luminex, TX, USA). Mouse glomerular mesangial cells were seeded at 1.0 × 10^6^ cells per 6-well plate in 2 mL DMEM/F-12 medium containing 5% FBS, incubated for 6 h, then pretreated with different concentrations (2, 10, and 50 μg/mL) of ECE for 1 h. Then, MGO was added for 24 h to induce apoptosis. After removing the medium, the cells were washed with cold HBSS, diluted to 500 cells/μL, resuspended in DMEM/F-12 medium, added with the Muse™ Annexin V & Dead Cell reagent, incubated for 20 min at room temperature in the dark, and analyzed using a MUSE cell analyzer (Merck Millipore, Sydney, Australia). Results are expressed as the percentage of total apoptotic cells (early and late apoptotic cells).

### 4.11. Western Blotting

The pretreated cells were scraped and proteins were extracted using PRO-PREP for Cell/Tissue (iNtRON Biotechnology, Seongnam-si, Korea) containing 1% protease and phosphatase (Thermo Fisher Scientific, Rockford, IL, USA). First, protein concentration was measured using a DC Protein Assay (Bio-Rad, Hercules, CA, USA). After a metal bath at 100 °C for 5 min, proteins were cooled with ice. The remaining protein samples were stored at −80 °C. The diluted protein samples were performed to gel electrophoresis using Any kD Mini-PROTEAN TGX Stain-Free Gels at 200 V, and transferred to polyvinylidene difluoride membranes using the Trans-Blot Turbo RTA Transfer Kit (Bio-Rad, Hercules, CA, USA). The membranes were blocked using 5% skim milk in Tris-buffered saline with 0.1% Tween-20 buffer for 1 h. Then, the membrane was incubated with primary antibodies (1:1000) at 4 °C for 24, followed by HRP-labeled goat anti-rabbit IgG or goat anti-mouse IgG (1:5000) for 1 h at 23 °C. Finally, the membranes were exposed to WesternSure PREMIUM Chemiluminescent Substrate (LI-COR, Lincoln, NE, USA). Luminescence was detected using the ChemiDoc MP Imaging System (Bio-Rad, Hercules, CA, USA).

### 4.12. Statistical Analysis

All experiments were independently performed in triplicate, and data are presented as mean ± standard deviation (*n* = 3). Data were statistically assessed by one-way analysis of variance (ANOVA) using GraphPad Prism ver. 9 (GraphPad Software, Inc., San Diego, CA, USA). Significant difference was performed followed by the Tukey’s test (*p* < 0.05).

## 5. Conclusions

In conclusion, it was confirmed that phlorotannin-rich *E. cava* extract (ECE) has anti-glycation ability, through the inhibition of AGE formation, inhibition of AGE-collagen cross-linking formation, and breaking of AGE-collagen cross-links. ECE plays a role in effectively protecting mouse glomerular mesangial cells by reducing MGO-induced cytotoxicity and oxidative stress. In addition, ECE pretreatment prevented MGO-mediated renal cell damage by downregulating the RAGE, and apoptosis-related protein expression and upregulating the Nrf2/ARE signaling pathway. These findings suggest that *E. cava* may be a powerful natural agent for the prevention and management of AGEs-related diabetic nephropathy.
